# Artificial Intelligence in Plastic Surgery: A Bibliometric and Visual Analysis of the 100 Most-Cited English-Language Publications

**DOI:** 10.1093/asjof/ojag148

**Published:** 2026-07-11

**Authors:** Jonathan Mokhtar, Meera Hallak, Michel Gabriel Cazenave, Lucas Kreutz-Rodrigues, Krishna S Vyas, Curtis L Cetrulo, Alexandre G Lellouch

## Abstract

Artificial intelligence (AI) is fundamentally transforming the landscape of plastic surgery, yet the structural architecture of its most influential scholarship has not been systematically characterized. This study presents a bibliometric and visual analysis of the 100 most-cited English-language AI publications in plastic surgery. Scopus was searched from database inception through January 15, 2026. Eligible articles underwent dual independent screening in Covidence, and the 100 most-cited publications were analyzed using R (v4.4.1) and VOSviewer (v1.6.18) for citation metrics, authorship networks, geographic and institutional contributions, journal distribution, and thematic categorization. Of the 3827 retrieved records, 357 met the full inclusion criteria, and the top 100 were identified. These articles collectively received 2701 citations (average: 27.01 ± 22.19), with a marked post-2022 publication surge accounting for 79% of the studies, with 2024 contributing to 34% of that share. Patient education and large language model–based consultation constituted the dominant thematic cluster (35%), followed by ethical and governance considerations (17%) and outcomes prediction and risk modeling (13%). Aesthetic surgery represented the most prolific specialty (34%), with craniofacial and breast reconstruction each contributing to 21%. Aesthetic Plastic Surgery (*n* = 16) and the Aesthetic Surgery Journal (*n* = 10) led journal representation. The United States dominated global scholarly output, whereas Peninsula Health in Australia emerged as the most impactful institution, with Rozen and Seth jointly leading the analysis as top authors. This analysis demonstrates the intellectual topography of AI scholarship in plastic surgery, identifying prevailing thematic concentrations and underexplored domains, and offering a contemporary framework for future research priorities and international collaboration.

Level of Evidence: 3 (Therapeutic)

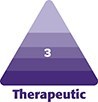

Artificial intelligence (AI) has rapidly evolved from a conceptual innovation to an operational tool across multiple surgical specialties. Within plastic and reconstructive surgery—a specialty characterized by the integration of aesthetic, microsurgical, and craniofacial principles with patient-centered outcomes—AI technologies are increasingly incorporated into clinical decision making, surgical planning, education, and research.^1^ Broadly, AI refers to algorithm-driven computational systems that analyze high-dimensional clinical and imaging data to enhance surgical planning, predict outcomes, and improve patient care.^2^ Because AI continues to influence plastic surgery practice and scholarship, the amount of literature examining its applications has grown substantially, reflecting the specialty's expanding engagement with data-driven methodologies.

Despite the accelerating growth of AI-related publications in plastic surgery, patterns of influence within the existing literature remain incompletely defined. Because the volume of research within this field increases, identifying the most impactful contributions, leading institutions, and dominant thematic areas becomes essential for understanding how the field is evolving. Bibliometric analyses provide a systematic framework for evaluating scientific productivity, citation impact, collaboration networks, and keyword trends, thereby offering insights into both influential research lines and emerging trends.^3^

Although individual reviews have explored AI applications in the specialty, a focused bibliometric analysis of the most-cited publications within this domain remains limited. In contrast, bibliometric analyses of AI have been conducted across numerous medical and surgical disciplines, including general surgery, orthopedics, radiology, and oncology, where they have helped identify shifts in research priorities, patterns of institutional leadership, and collaborative networks.^4-7^ The absence of a comparable, specialty-specific mapping within plastic and reconstructive surgery represents a critical gap in understanding how AI research has developed, where scholarly attention is concentrated, and which areas are emerging as future priorities.

Therefore, the authors aim to conduct a bibliometric and visual analysis of the top 100 most-cited English-language publications on AI within plastic surgery. By evaluating citation metrics, authorship patterns, institutional and geographic contributions, journal distribution, and thematic keyword networks, this analysis seeks to map the structure and development of AI-related research within the field, identifying prevailing themes, key contributors, and emerging areas of focus. In doing so, this study provides a structured foundation for understanding the trajectory of scholarly attention within plastic surgery and offers a clear framework to guide future research, collaboration, and clinical innovation.

## METHODS

### Search Strategy

A comprehensive literature search was conducted on January 15, 2026, by an individual author using the Scopus (Elsevier, Amsterdam, the Netherlands) database to identify all articles on the application of AI in plastic, reconstructive, and aesthetic surgery. The search strategy was developed with the aid of a medical librarian and encompassed a wide range of keywords relevant to both AI and plastic surgery such as “artificial intelligence,” “machine learning”, “large language models”, “plastic surgery,” “aesthetic surgery,” and “craniofacial surgery,” with the full search strategy in Appendix A. Scopus was selected as the search platform because it represents the most comprehensive abstract and citation database available for biomedical and surgical research, indexing ∼28,000 peer-reviewed journals—substantially more than the Web of Science (Clarivate Analytics, Philadelphia, PA) Core Collection (∼22,000 journals) and the subject-restricted PubMed (National Library of Medicine, Bethesda, MD), and provides ∼20% greater citation coverage than Web of Science (Clarivate Analytics), making it the preferred database for citation-based bibliometric analyses.^8,9^ Additionally, the use of a single database was informed by landmark bibliometric analyses in the specialty.^10-12^

To ensure a rigorous and transparent screening process, the initial studies identified on the Scopus (Elsevier) database were imported into Covidence (Veritas Health Innovation, Melbourne, Australia), a specialized web-based systematic review management platform.^13^ To minimize bias, 2 independent investigators screened each article at both the title/abstract and full-text stages. Disagreements regarding inclusion were resolved through consensus or arbitration by a third senior author. Articles were included if they met the following criteria: (1) original research or review articles; (2) direct relevance to the application of AI in plastic, reconstructive, or aesthetic surgery; and (3) publication in the English language. No restrictions were placed on the publication date to ensure a comprehensive historical analysis.

### Data Extraction

For each of the 100 selected articles, a comprehensive set of bibliometric data was directly exported from the database into a single unified comma-separated values (CSV) file. This exported dataset included the following variables for each article: (1) basic publication details (title, year, and journal); (2) citation metrics (total citations); (3) authorship details (author names and affiliations); (4) geographic information (country of origin); (5) institutional affiliations; (6) document characteristics (document type and open-access status); and (7) funding information (funding details and sources). Citation density was subsequently calculated by dividing the total number of citations by the number of years since publication (up to 2025). The direct export method ensured the accuracy and completeness of the data as recorded in the database.

### Thematic Categorization

To systematically characterize the intellectual landscape of the top 100 most-cited English-language publications, each article was assigned to a primary thematic category reflecting its principal AI application domain. Categories were defined a priori by 2 independent investigators based on established lines of AI research in medicine for the full text of each included article. In cases of disagreement, a third senior investigator was consulted, and a final category was assigned by consensus. The final taxonomy comprised the following domains: (1) patient education and large language model (LLM)-based consultation; (2) ethical, legal, and governance considerations; (3) outcomes, prediction, and risk modeling; (4) diagnosis, imaging, and anatomical analysis; (5) aesthetic assessment and objective metrics; (6) education and training (surgeon focused); (7) robotics and automation; (8) general overviews; and (9) basic science and genetics. Each article was assigned to a single primary category; however, when a study addressed multiple domains, classification was determined by the principal focus as defined in the study objectives.

### Level of Evidence Assessment

To evaluate the quality of evidence, each of the 100 articles was assigned an evidence level according to the 2011 American Society of Plastic Surgeons (ASPS) evidence rating.^14^ This classification was performed by 2independent authors who examined the title, abstract, and full text of each article to determine the study design. Articles were categorized as Level I (high-quality multicenter or single-center randomized controlled trials with adequate evidence or systematic review of these studies), Level II (lesser-quality randomized controlled trial; prospective cohort or comparative study; or systematic review of these studies), Level III (retrospective cohort or comparative study or systematic review of these studies), Level IV (case series with pre/posttest or only posttest), or Level V (expert opinion developed through consensus process; case report or clinical example; or evidence based on physiology, bench research or “first principles”). Any disagreements in classification were resolved by a third senior author.

### Statistical Analysis

Descriptive and bibliometric analyses were performed using R software (version 4.4.1; R Foundation for Statistical Computing, Vienna, Austria).^15^ Continuous variables were summarized using means, standard deviations, ranges, medians, and interquartile ranges. Categorical variables were reported as frequencies and percentages (%).

For network analyses and visualization, VOSviewer (version 1.6.18; Center for Science and Technology Studies, Leiden University, the Netherlands) was employed.^16^ This tool was used to generate bibliometric maps to visualize the collaborative and conceptual structure of the research field. Specifically, we conducted (1) co-authorship analysis of authors to identify influential research groups; (2) co-occurrence analysis of countries to map international collaboration networks, where the strength of the link represents the frequency of co-authorship between countries; and (3) co-occurrence analysis of author-defined keywords to identify major research themes and emerging trends. In the network maps, the size of each node is proportional to the frequency of occurrence, and the thickness of the connecting lines indicates the strength of the relationship between nodes.

## RESULTS

**Figure 1.**
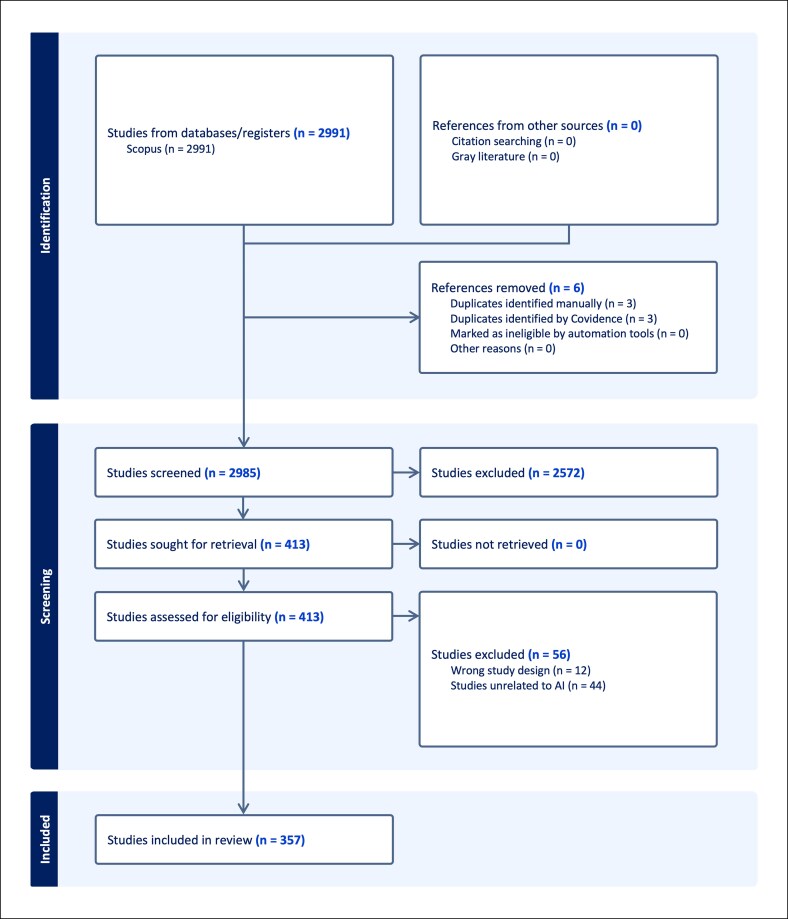
PRISMA flow diagram illustrating the database used, the studies retrieved for title and abstract screening, full-text screening, and final inclusion of studies with reasons for exclusion in the present study. AI, artificial intelligence.

The initial systematic database search yielded 2991 publications after excluding non-English articles and irrelevant document types (original number of publications from the database before importing into the screening platform: 3827). As shown in Figure 1, the title and abstract screening resulted in 413 articles sought for full-text retrieval. Full-text screening excluded 56 articles (including 44 that were unrelated to AI in plastic surgery), resulting in 357 articles that met all inclusion criteria. The citation data for these 357 articles were imported into VOSviewer (version 1.6.18; Center for Science and Technology Studies, Leiden University, the Netherlands), which identified the 100 most-cited publications for final bibliometric analysis. Of the 100 included studies, 86 were original research articles, and 14 were reviews; the full bibliographic information is provided in Appendix B.

### Publication Year and Citation

**Figure 2.**
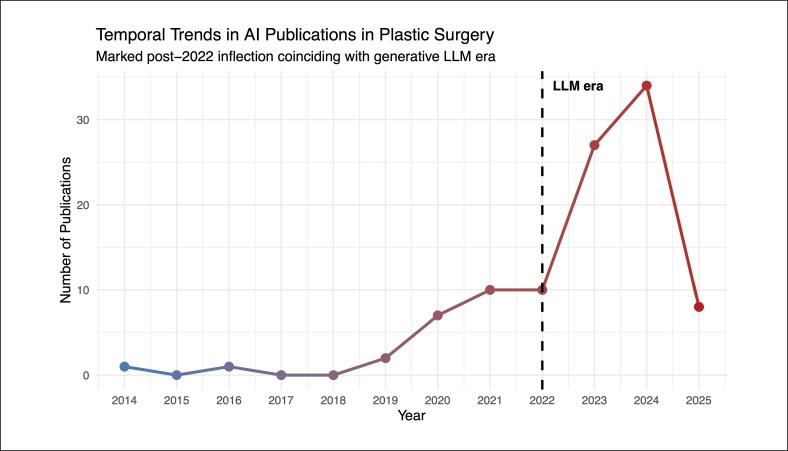
Temporal trends in English-language AI publications in plastic surgery (2014-2025). A sharp increase in publications is observed after 2022, corresponding to the generative AI and LLM era. AI, artificial intelligence; LLM, large language model.

The 100 most-cited English-language AI articles in plastic surgery collectively received 2701 citations. The citation counts per article ranged from 9 to 139, with an average of 27.01 (22.19) and a citation density of 6 citations per year (range, 1.38-34.75). This body of literature is relatively recent, with publications dating from 2014 to 2025. A significant acceleration in research output was observed from 2022 onward, with this period accounting for 79% of the top-cited articles (Figure 2). Regarding publication years, 2024 was the most productive, accounting for 34% of articles, followed by 2023 at 27%.

The most-cited article in the dataset was published in 2023 by Xie et al, titled “Aesthetic Surgery Advice and Counselling from Artificial Intelligence: A Rhinoplasty Consultation with ChatGPT,” published in Aesthetic Plastic Surgery, which accumulated 139 total citations and a citation density of 34.75 per year—the highest in the cohort.^17^ The second most-cited article, by Kanevsky et al, titled “Big Data and Machine Learning in Plastic Surgery: A New Frontier in Surgical Innovation,” published in Plastic and Reconstructive Surgery, accumulated 115 citations and a citation density of 10.45 per year.^18^ The third most-cited article was by Seth et al, titled “Evaluating Chatbot Efficacy for Answering Frequently Asked Questions in Plastic Surgery: A ChatGPT Case Study Focused on Breast Augmentation,” published in the Aesthetic Surgery Journal, with 97 citations and a citation density of 24.25 per year.^19^

Notably, among the 20 most-cited articles shown in Table 1, 40% were published in 2023 alone, highlighting that year as one of the most productive periods for highly cited AI research in the discipline.^17-36^ The year 2020 was the second-most represented year among the top 20 highest-cited articles, contributing 30% of the articles, with a focus on machine learning (ML) applications in rhinoplasty, facelift surgery, and microsurgical reconstruction.

**Table 1.** Characteristics of the Top 20 Most-Cited Articles Among the Top 100 Most-Cited English-Language Publications on AI in Plastic Surgery

Ranking	Title	First author	Year	Journal	TC	TC per year	Normalized TC	IF (2024)
**1**	Aesthetic surgery advice and counselling from artificial intelligence: a rhinoplasty consultation with ChatGPT	Xie et al^17^	2023	Aesthetic Plastic Surgery	139	34.75	4.15	2.8
**2**	Big data and machine learning in plastic surgery: a new frontier in surgical innovation	Kanevsky et al^18^	2016	Plastic and Reconstructive Surgery	115	10.45	1.00	3.4
**3**	Evaluating chatbot efficacy for answering frequently asked questions in plastic surgery: a ChatGPT case study focused on breast augmentation	Seth et al^19^	2023	Aesthetic Surgery Journal	97	24.25	2.90	3.9
**4**	Personalized assessments of craniosynostosis via statistical shape modeling	Mendoza et al^20^	2014	Medical Images Analysis	95	7.31	1.00	11.8
**5**	Expanding cosmetic plastic surgery research with ChatGPT	Gupta et al^21^	2023	Aesthetic Surgery Journal	86	21.50	2.57	3.9
**6**	Diagnose Parkinson's disease and cleft lip and palate using deep convolutional neural networks evolved by IP-based chimp optimization algorithm	Chen et al^23^	2022	Biomedical Signal Processing and Control	65	13.00	2.40	4.9
**7**	Evaluation of online artificial intelligence-generated information on common hand procedures	Crook et al^22^	2023	Journal of Hand Surgery	64	16.00	1.91	2.1
**8**	Machine learning for predicting complications in head and neck microvascular free tissue transfer	Formeister et al^24^	2020	The Laryngoscope	50	7.14	1.29	2.47
**9**	Applied deep learning in plastic surgery: classifying rhinoplasty with a mobile app	Borsting et al^25^	2020	Journal of Craniofacial Surgery	47	6.71	1.21	1.0
**10**	Exploring the role of a large language model on carpal tunnel syndrome management: an observation study of ChatGPT	Seth et al^26^	2023	Journal of Hand Surgery	46	11.50	1.37	2.1
**11**	Artificial intelligence applications and ethical challenges in oral and maxillo-facial cosmetic surgery: a narrative review	Rokhshad et al^27^	2023	Maxillofacial Plastic and Reconstructive Surgery	44	11.00	1.31	2.8
**12**	Optimizing ophthalmology patient education via ChatBot-generated materials: readability analysis of AI-generated patient education materials and the American Society of Ophthalmic Plastic and Reconstructive Surgery brochures	Eid et al^28^	2024	Ophthalmic Plastic and Reconstructive Surgery	43	14.33	2.38	1.3
**13**	Clinical application of artificial intelligence and machine learning in children with cleft lip and palate––a systematic review	Huqh et al^29^	2022	International Journal of Environmental Research and Public Health	41	8.20	1.51	3.1
**14**	Making the subjective objective: machine learning and rhinoplasty	Dorfman et al^30^	2020	Aesthetic Surgery Journal	41	5.86	1.06	3.9
**15**	Automatic detection of perforators for microsurgical reconstruction	Mavioso et al^31^	2020	The Breast	41	5.86	1.06	7.9
**16**	Machine learning applied to registry data: development of a patient-specific prediction model for blood transfusion requirements during craniofacial surgery using the pediatric craniofacial perioperative registry dataset	Jalali et al^32^	2021	Anesthesia and Analgesia	39	6.50	1.45	4.0
**17**	Using generative artificial intelligence tools in cosmetic surgery: a study on rhinoplasty, facelifts, and blepharoplasty procedures	Lim et al^33^	2023	Journal of Clinical Medicine	37	9.25	1.11	2.9
**18**	Facelift surgery turns back the clock: artificial intelligence and patient satisfaction quantitate value of procedure type and specific techniques	Gibstein et al^34^	2020	Aesthetic Surgery Journal	36	6.00	1.34	3.9
**19**	Use of simulation in plastic surgery training	Agrawal et al^35^	2020	Plastic and Reconstructive Surgery Global Open	36	5.14	0.93	1.8
**20**	ChatGPT in plastic and reconstructive surgery	Sharma et al^36^	2023	Indian Journal of Plastic Surgery	35	8.75	1.05	1.5

AI, artificial intelligence; IF, impact factor; TC, total citations.

### Topics, Keywords, and Categories

Analysis of the thematic landscape revealed that most published articles addressed clinical and translational applications of AI. The most prevalent broad topic was patient education/LLM consultation, which accounted for 35% of articles. The second most common theme was ethical, legal, and governance considerations, representing 17% of articles. Outcomes prediction and risk modeling constituted the third largest category with 13% of the published articles, encompassing ML models designed to predict surgical complications, blood transfusion requirements, flap failure, and patient-reported outcomes. The full distribution of thematic categories across the top 100 articles is presented in Table 2.

**Table 2.** Distribution of the Top 100 Most-Cited English-Language Artificial Intelligence Articles in Plastic Surgery by Broad Topic Area

Broad topic	No. of publications
**Patient** e**ducation/LLM** c**onsultation**	35
**Ethical/**l**egal/**g**overnance**	17
**Outcomes/**p**rediction/**r**isk** m**odeling**	13
**Diagnosis/**i**maging/**a**natomical Analysis**	10
**Aesthetic** a**ssessment/**o**bjective** m**etrics**	9
**Education and** t**raining (**s**urgeon focused)**	6
**Robotics/**a**utomation**	5
**General/**o**verview**	3
**Basic** s**cience/**g**enetics**	2

LLM, large language model.

From a subspecialty perspective, aesthetic surgery was the most-represented domain, accounting for 34% of published articles. This was followed by craniofacial/cleft surgery and breast surgery and reconstruction, each accounting for 21% of articles, respectively. A full breakdown of subspecialty representation across the included studies is presented in Table 3.

**Table 3.** Distribution of the Top 100 Most-Cited English-Language Artificial Intelligence Articles in Plastic Surgery by Subspecialties

Subspecialty	No. of publications
**Aesthetic** s**urgery**	34
**Craniofacial/**c**left**	21
**Breast** s**urgery and** r**econstruction**	21
**Oral and** m**axillofacial**	8
**General** p**lastic** s**urgery**	7
**Hand** s**urgery**	5
**Microsurgery**	4

Assessment of methodological quality using ASPS's evidence-based hierarchy revealed that most included studies were dominated by Level IV evidence (62%), comprising case series, pre- and posttest designs, and single-cohort observational studies. Level V evidence was the second largest category, accounting for 28% of the included articles. The full distribution of evidence levels of the remaining articles is presented in Table 4.

**Table 4.** Level of Evidence Classification of the Top 100 Most-Cited Articles According to the American Society of Plastic Surgeons’ Evidence-Based Hierarchy

Level of evidence	Type of study	No. of publications
**I**	High-quality, multicenter or single-center, randomized controlled trial with adequate power; or systematic review of these studies	1
**II**	Lesser-quality, randomized controlled trial; prospective cohort or comparative study; or systematic review of these studies	3
**III**	Retrospective cohort or comparative study; case–control study; or systematic review of these studies	6
**IV**	Case series with pre/posttest or only posttest	62
**V**	Expert opinion developed through consensus process; case report or clinical example; or evidence based on physiology, bench research, or “first principles”	28

**Figure 3.**
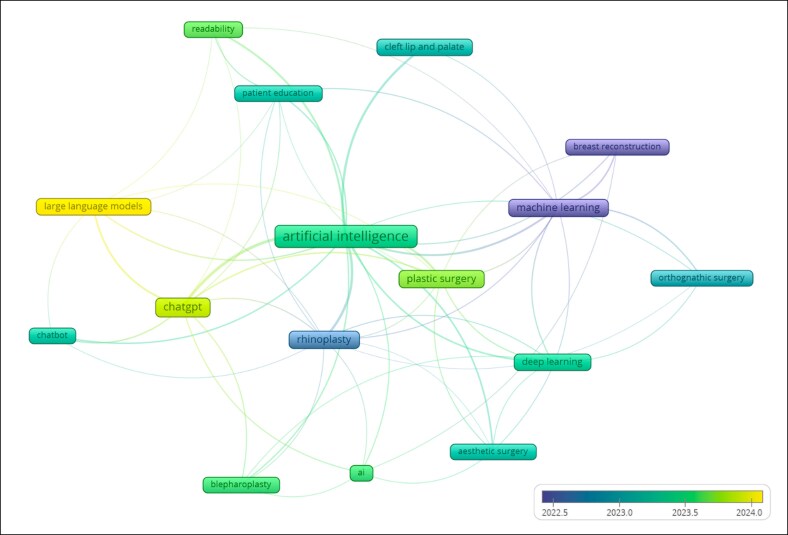
Keyword co-occurrence network illustrating thematic relationships among the top 100 most-cited English-language AI publications in plastic surgery. Nodes represent keywords, with node size proportional to frequency, and links indicate co-occurrence between terms across the included studies. The color gradient reflects the average publication year, highlighting the temporal emergence of topics such as LLMs and ChatGPT (OpenAI, San Francisco, CA) in recent literature. AI, artificial intelligence; LLM, large language model.

The keywords co-occurrence network revealed a well-defined intellectual core on “artificial intelligence” as the dominant node, with the strongest co-occurrence linkages observed with “machine learning,” “deep learning,” “plastic surgery,” “rhinoplasty,” “cleft lip and palate,” “breast reconstruction,” and “orthognathic surgery.” A distinct cluster of more recently emergent keywords, highlighted in yellow-green tones corresponding to 2023-2024, included “ChatGPT,” “large language models,” “chatbot,” “patient education,” “readability,” and “blepharoplasty” (Figure 3). The temporal gradient of the network confirms that although foundational AI and ML concepts anchored the field's early development, there has been a shift toward generative AI and conversational interfaces in surgical practice after 2022.

### Journal Analysis

The 100 most-cited articles were distributed across 41 unique journals, of which 63.4% of them were open-access journals. Plastic and Reconstructive Surgery achieved the highest mean citation rate in the cohort at 37.2 citations per article (6 articles; 233 total citations). The Aesthetic Surgery Journal ranked second by mean citation rate at 34.6 citations per article (10 articles; 346 total citations). Aesthetic Plastic Surgery led by publication volume with 16 articles (420 total citations; mean citations per article of 26.25). The full journal citation metrics are presented in Table 5.

**Table 5.** Journals Contributing the Highest Number of Publications Among the Top 100 Most-Cited English-Language Artificial Intelligence Articles in Plastic Surgery

Ranking	Journal	No. of publications	TC	Average citations per article	IF 2024	JCR	Publication year start
**1**	Aesthetic Plastic Surgery	16	420	26.25	2.8	Surgery Q1	2021
**2**	Aesthetic Surgery Journal	10	346	34.6	3.9	Surgery Q1	2020
**3**	Journal of Craniofacial Surgery	8	176	22	1	Surgery Q3	2020
**4**	Plastic and Reconstructive Surgery	6	223	37.2	3.4	Surgery Q1	2016
**5**	Plastic and Reconstructive Surgery—Global Open	6	152	25.3	1.8	Surgery Q2	2020
**6**	Facial Plastic Surgery	4	66	16.5	1.4	Surgery Q2	2022
**7**	Journal of Clinical Medicine	4	105	26.25	2.9	Medicine, General and Internal Q1	2023
**8**	Cleft Palate Craniofacial Journal	3	63	21	1.3	Surgery Q1	2024
**9**	Journal of Plastic, Reconstructive and Aesthetic Surgery	3	40	13.3	2.4	Surgery Q1	2023
**10**	Journal of Plastic, Reconstructive and Aesthetic Surgery Open	3	48	16	1.8	Surgery Q2	2024

IF, impact factor; JCR, journal citation reports; TC, total citations.

### Country, Institution, and Author

The top 100 most-cited articles originated from 29 contributing countries. The United States was the dominant contributor, accounting for 45 articles and 1205 total citations—representing 44.6% of all citations among studies, with an average citation per article of 26.8 (18.6). Australia ranked second with 10 articles and 445 citations, achieving the highest average citation rate among all contributing countries at 44.5 (41.2) citations per article. China ranked third with 8 articles and 226 total citations (average: 28.2 [17.1]), contributing substantially to craniofacial and cleft-related AI research (Table 6).

**Table 6.** The Top 5 Countries Contributing the Most Publications to the Top 100 Most-Cited English-Language Artificial Intelligence Articles in Plastic Surgery

Rank	Country	No. of publications	TC	Mean citations per article
**1**	USA	45	1205	26.8 (18.6)
**2**	Australia	10	445	44.5 (41.2)
**3**	China	8	226	28.2 (17.1)
**4**	South Korea	6	91	15.2 (5.1)
**5**	Germany	4	104	26 (15.2)

TC, total citations.

**Figure 4.**
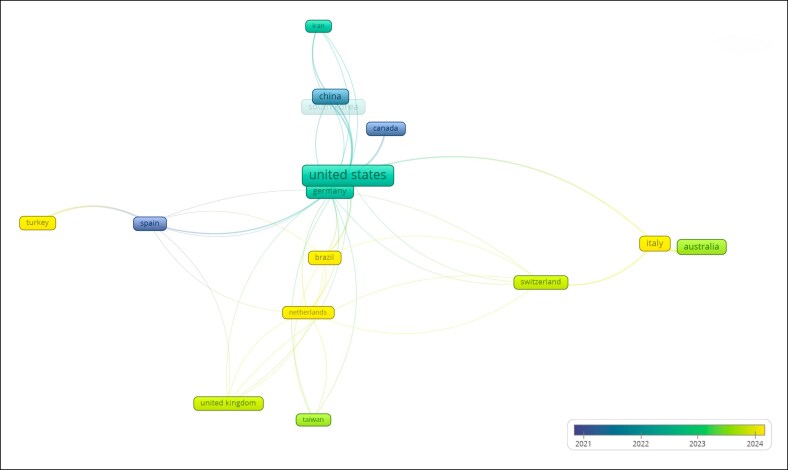
Country collaboration co-occurrence network illustrating thematic relationships among the top 100 most-cited English-language artificial intelligence publications in plastic surgery. Nodes represent countries, with node size proportional to frequency, and links indicate co-occurrence between countries across the included studies. The color gradient reflects the average publication year, highlighting the increasing prevalence of contributions among different countries.

The country co-occurrence network revealed that the Germany–US axis was the most frequent bilateral collaboration, co-authoring 6 articles. The Australia–Italy partnership was the second most productive bilateral collaboration with 5 co-authored articles, and concentrated on LLM performance evaluation across a range of aesthetic and reconstructive procedures. The US–Brazil, US–South Korea, and US–China pairings each contributed 3 co-authored articles, reflecting the US's role as the central hub of the global collaboration network and its capacity to anchor multi-continental research partnerships across diverse subspecialty domains (Figure 4).

Among the 211 contributing institutions, the Department of Plastic Surgery, Peninsula Health, Victoria, Australia, emerged as the most impactful single institution, contributing 8 publications as the primary affiliation (9 total) and accumulating 414 total citations with an average of 46 (43.4) citations per article and a citation density of 12.25 (10.34). Monash University, Melbourne, Australia, ranked second with 5 publications and 257 total citations (51.4 [50.5] average citations per article; 13.17 [12.4] citation density). The Plastic Surgery Unit, University of Siena (Italy), ranked third with 5 publications and 121 total citations (24.2 [9.5] average citations per article; 7.92 [1.77] citation density), representing the most-cited European institutional contributor (Table 7).

**Table 7.** The Top 10 Institutions Contributing the Most Publications to the Top 100 Most-Cited English-Language Artificial Intelligence Articles in Plastic Surgery

Rank	Institution	City, country	No. of publications	No. of first affiliations	TC	Citations per article	Citation density
**1**	Department of Plastic Surgery, Peninsula Health	VIC, Australia	9	8	414	46 (43.4)	12.25 (10.34)
**2**	Monash University	Melbourne, VIC, Australia	5	1	257	51.4 (50.5)	13.17 (12.4)
**3**	Plastic Surgery Unit, Dipartimento di Scienze Mediche, Chirugiche e Neuroscienze	Siena, SI, Italy	5	0	121	24.2 (9.5)	7.92 (1.77)
**4**	Division of Plastic Surgery, Mayo Clinic	Jacksonville, FL	3	3	71	23.7 (10.4)	7.36 (2.88)
**5**	Department of Plastic and Reconstructive Surgery, The University of Tokyo Hospital	Tokyo, Japan	3	3	40	13.3 (2.5)	4.44 (0.84)
**6**	Melbourne Medical School	Melbourne, Australia	2	0	72	36 (14.1)	9 (3.54)
**7**	UCI School of Medicine	Irvine, CA	2	1	64	32 (21.2)	5.06 (2.34)
**8**	Microsoft Corporation	Redmond, WA	2	0	61	30.5 (7.8)	5.08 (1.3)
**9**	Department of General Surgery, Sinai Hospital of Baltimore	Baltimore, MD	2	0	55	27.5 (4.9)	9.88 (3.01)
**10**	Department of Plastic Surgery, Vanderbilt University Medical Center	Nashville, TN	2	2	55	27.5 (4.9)	9.88 (3.01)

TC, total citations.

**Figure 5.**
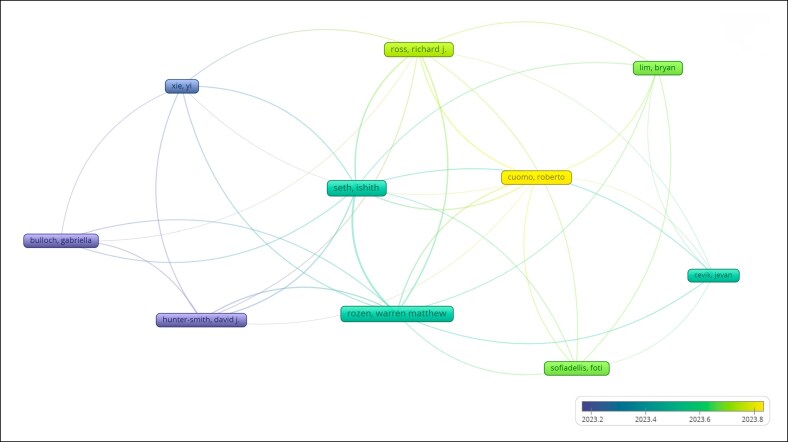
Author collaboration co-occurrence network illustrating thematic relationships among the top 100 most-cited English-language artificial intelligence publications in plastic surgery. Nodes represent author names, with node size proportional to frequency and links indicating co-occurrence between authors within the included studies.

Among the 571 unique authors contributing to the top 100 most-cited articles, Rozen and Seth (both at Peninsula Health) jointly led the analysis with 10 publications each and 445 total citations (44.5 [41.2] mean citations per article and a citation density of 12.1 [9.8]). Ross (Peninsula Health) ranked third with 7 publications and 357 total citations (51 [47.9] average citations per article and a citation density of 14.1 [11.1]). Interestingly, Xie (Peninsula Health), despite contributing only 4 publications, achieved the highest mean citation count in the entire study at 78.25 (49.4) citations per article and a citation density of 20.2 (11.6), driven principally by the most-cited article in the dataset (Table 8, Figure 5).

**Table 8.** The Top 10 Most Relevant Authors Contributing the Most Publications to the Top 100 Most-Cited English-Language Artificial Intelligence Articles in Plastic Surgery

Rank	Author	Institution, country	No. of publications	TC	Average citations per article	Citation density
**1**	Rozen, Warren Matthew	Peninsula Health, Australia	10	445	44.5 (41.2)	12.1 (9.8)
**2**	Seth, Ishith	Peninsula Health, Australia	10	445	44.5 (41.2)	12.1 (9.8)
**3**	Ross, Richard J.	Peninsula Health, Australia	7	357	51 (47.9)	14.1 (11.1)
**4**	Hunter-Smith, David J.	Monash University, Australia	5	339	67.8 (48.7)	17.5 (11.7)
**5**	Cuomo, Roberto	University of Siena, Italy	5	121	24.2 (9.5)	7.9 (1.8)
**6**	Xie, Yi	Peninsula Health, Australia	4	313	78.25 (49.4)	20.2 (11.6)
**7**	Bulloch, Gabriella	The University of Melbourne, Australia	3	169	56.3 (36.6)	14.1 (9.2)
**8**	Sofiadellis, Foti	Peninsula Health, Australia	3	76	25.3 (10.1)	7.4 (1.6)
**9**	Lim, Bryan	Peninsula Health, Australia	3	76	25.3 (10.1)	7.4 (1.6)
**10**	Forte, Antonio Jorge	Mayo Clinic, Jacksonville, FL	3	71	23.7 (10.4)	7.4 (2.9)

TC, total citations.

### Funding Information

Twenty-two percent of articles in this dataset declared formal external funding support, whereas the remaining 78% were conducted without declared funding. The most frequently cited funding body was the National Institute of Dental and Craniofacial Research, which supported 6 funded articles. The National Natural Science Foundation of China was the second most common funder, appearing across 4 articles. The Ministry of Health and Welfare of South Korea and the Korea Health Industry Development Institute jointly supported 3 and 2 articles, respectively. The remaining studies were funded by institutions and foundations across the United States, Europe, and Asia.

A subanalysis comparing citation metrics between funded and unfunded articles revealed no statistically significant difference in citation impact. Funded articles had an average citation count of 24.6 (16.1) compared with 27.7 (23.7) for unfunded articles (Mann–Whitney *U* = 788.0, *P* = .563). There was no association between funding status and open-access publications, as only 5 of the 22 funded studies appeared exclusively in open-access journals.

## DISCUSSION

In this bibliometric and visual analysis of the 100 most-cited English-language publications on AI in plastic and reconstructive surgery, we document a significant wave of publications, demonstrating that the field has undergone a rapid, concentrated expansion over the past decade, with the most-cited work emerging after 2022. It is important to contextualize this finding: the post-2022 publication surge observed here is not unique to plastic surgery but reflects a broad, field-agonistic acceleration in AI research following the widespread public release of LLMs. Similar inflection points have been documented across multiple other specialties, suggesting that the trend reflects the general maturation of AI technology rather than a specialty-specific development.^4-6^ What is meaningful for plastic surgery is the thematic configuration of this growth: the concentration in patient-facing LLM applications, aesthetic subspecialties, and ethical governance, which may reflect the specialty's particular emphasis on direct patient communication, shared decision making, and aesthetic outcome visualization—domains in which LLMs are particularly important for evaluation, as well as the relative accessibility of LLM-based study designs. Collectively, these patterns suggest that AI is rapidly transitioning from a conceptual innovation to a practical framework that is reshaping research priorities, clinical workflows, and future technological development within the specialty.

An additional lens for interpreting these thematic patterns is to consider not merely what authors are publishing but what the citing community appears to prioritize: which types of studies are generating scholarly engagement? Examining the citation networks within this dataset reveals that the most-cited articles cluster around 3 functionally distinct citation-attracting categories: (1) patient-facing LLM evaluations, which are heavily cited by subsequent chatbot comparison studies, citing them as benchmarks; (2) ethical and governance frameworks, which are cited disproportionately by clinical and regulatory papers seeking precedent for AI oversight; and (3) outcome-prediction models using large registry datasets, which attract citations from clinical investigators seeking risk-stratification tools. This citation-purpose grouping suggests that the community may place higher scholarly value on reproducible methodological frameworks and clinically actionable risk models, although these categories are outnumbered by patient education publications in raw counts. Another notable finding in the present analysis is the distribution of subspecialties: 3 common subspecialties (aesthetic, craniofacial, and breast reconstruction) followed the trajectory of previous non-AI-related bibliometric analyses in the field. This potentially highlights the advancement of AI-driven research along the same subspecialty lines that have historically dominated the plastic surgery literature, suggesting that AI adoption may be reinforcing, rather than reshaping existing patterns of scholarly focus within the field.^11,12,37,38^

The development of AI research in plastic surgery reflects a broader progression within the specialty's literature. Early discussions focused primarily on big data and ML, particularly in predictive analytics and surgical outcome modeling.^39^ These early studies helped introduce AI concepts into surgical research and demonstrated that computational approaches could be applied to complex surgical datasets. Over time, these ideas have expanded into more advanced clinical applications across several subspecialties. Recent work shows how AI is being used for tasks such as surgical planning and knowledge retrieval in craniofacial surgery, including tools such as CASPER (developed by Cleveland Clinic, Cleveland, OH, in collaboration with Boston University and Case Western Reserve University), a retrieval-augmented generation (RAG) platform that allows surgeons to rapidly access diagnosis- and algorithm-related information synthesized from peer-reviewed open-access literature.^40^ Similarly, RAG-based tools such as MicroRAG (developed by Cleveland Clinic in collaboration with Boston University and Case Western Reserve University) support rapid access to evidence for microsurgical decision making, whereas LLM-based systems are increasingly evaluated for patient education and consultation in aesthetic surgery, reflecting a broader shift toward practical, clinician- and patient-facing AI applications.^41,42^

At the same time, there has been growing attention to the ethical considerations and responsible implementation of AI across multiple areas of the specialty, including aesthetic surgery, reconstructive microsurgery, and craniofacial surgery.^43-45^ Because AI systems increasingly influence diagnostic evaluation, surgical planning, and perioperative decision making, concerns have been raised about algorithmic bias, privacy risks associated with facial imaging data, and the need for transparency and explainability in AI-driven recommendations. These challenges are particularly relevant in craniofacial surgery, where pediatric patients are the focus and clinical decisions often rely on sensitive imaging data. Accordingly, recent work emphasizes the importance of clear regulatory oversight, local validation of AI systems, and governance frameworks to ensure that these technologies are implemented safely and responsibly.^43^

AI-based predictive models are also increasingly explored in reconstructive surgery, including breast reconstruction, where ML models are being developed to improve perioperative risk stratification and complication forecasting.^46^ For instance, recent work using large national surgical databases has demonstrated how ML models can identify factors associated with postoperative complications following autologous breast reconstruction, such as elevated BMI, prolonged operative time, and longer hospital stays.^46^ Rather than functioning as autonomous decision-making systems, these models primarily support surgeons by highlighting patient-specific risk patterns within large clinical datasets. By using large-scale clinical datasets, these models offer the potential to support more individualized risk assessment and improve preoperative counseling in complex reconstructive procedures.

Despite this progress, significant structural barriers continue to limit the responsible clinical integration of AI in plastic surgery. As Ozmen and Taub have identified, the primary modifiable barriers to implementation include limited AI literacy and fragmented clinician–data-science collaboration; an immature human–capital pipeline in which junior AI individuals lack senior sponsorship, protected research time, and misaligned institutional incentives that undervalue dataset curation, code sharing, and rigorous external validation.^43^ These barriers represent an organizational readiness gap rather than a modeling deficit, and addressing them will require embedding AI literacy across surgical training and continuing medical education, formalizing partnerships between clinical and engineering departments, and establishing protected time for data preparation and governance workflows. The geographic disparities in AI research output observed in the present analysis may therefore reflect not only differences in computational resources and data access but also fundamental differences in institutional readiness and collaborative infrastructure. This framing suggests that bibliometric leadership does not necessarily translate into clinical implementation readiness, and that countries with fewer publications may nonetheless be making important progress in responsible AI integration that is not yet captured by citation-based metrics.^47^

Bibliometric analyses have shaped the future of AI research in other medical and surgical specialties by identifying key trends, key contributors, and emerging areas of investigation. In orthopedic surgery and radiology, early applications of ML for imaging analysis and outcome prediction were followed by the rapid expansion of generative AI and clinical decision support systems as the technology matured.^5,6^ Bibliometric analyses in these fields have helped map the evolution of AI research and identify emerging areas of investigation, often guiding subsequent scholarly activity. For example, bibliometric studies examining AI research trends in orthopedic surgery have been followed by an increasing number of investigations exploring specific clinical applications of AI in musculoskeletal imaging, surgical planning, and outcome prediction.^48-50^ By providing a structured overview of research activity, these analyses allow investigators to identify high-impact topics and gaps in the literature, thereby guiding future research priorities.

Our findings suggest that AI research in plastic surgery is following a similar trajectory and that the present analysis may serve as a contemporary framework to guide future investigations, encourage collaboration, and support the continued growth of clinically relevant AI applications within the specialty. The specific actionable contributions of this analysis are 3-fold. First, by identifying the thematic configuration of the most-cited scholarship, this study enables investigators to suggest which areas are currently heavily presented in highly cited literature (such as LLM patient education evaluations, noting that citation frequency reflects scholarly interest rather than total publication volume, meaning a high citation count does not definitively equal overall topic saturation) vs which remain underexplored relative to clinical need (outcome prediction, surgical planning, and reconstructive decision support). Second, by mapping the geographic and institutional concentration of output, this study identifies potential collaboration targets for investigators seeking to build international research networks in AI plastic surgery. Third, by characterizing the distribution of evidence levels (dominated by Level IV and V studies), this analysis highlights the urgent need for higher-quality prospective designs and randomized evaluations as the field matures, because this predominance is a direct and expected consequence of the study designs, which are inherently exploratory and do not lend themselves to randomization.

Another notable finding from our analysis was the geographic distribution and collaboration patterns. The United States emerged as the leading contributor in total publications and citations in our analysis, consistent with its long-standing influence in plastic surgery research as demonstrated by previous bibliometric analyses.^10-12^ In addition to producing the most studies in the present analysis, the United States also served as a central hub for international collaborations, frequently partnering with institutions across Europe, Asia, and Australia. These collaboration networks suggest that US institutions play an important role in connecting research groups and facilitating multinational AI research efforts within the specialty. Interestingly, Australia demonstrated a disproportionately strong influence relative to its size, with Peninsula Health in Victoria contributing a particularly high number of influential publications. However, this elevated per-article citation rate likely reflects a small sample size of publications, concentrated authorship within a single institutional group, and strategic journal targeting, rather than a proportionally larger research contribution, in contrast to the country-level trends reported in non-AI bibliometric analyses.^10-12^ This concentration of output highlights how focused institutional research programs can generate substantial scholarly attention within emerging areas of surgical innovation, while also reflecting the need for caution when interpreting citation-based metrics from small national cohorts. At the same time, our findings from the 100 most-cited English language publications on AI in plastic surgery reveal relatively limited representation from several regions, particularly the Middle East and Africa, despite growing global interest in AI technologies. It is important to note, however, that this geographic pattern should be interpreted with caution, because the restriction to English-language publications may systematically underrepresent contributions from non-English-speaking regions, potentially inflating the apparent dominance of other countries and underestimating scholarly activity in these regions.

The regulatory landscape for AI in healthcare varies markedly across contributing countries and may partially explain observed geographic clustering. The United States, which led in both output and collaboration, operates within a relatively established FDA oversight framework for AI-enabled medical devices, potentially creating both a permissive environment for translational research and an incentive for regulatory-driven publications. China, the third largest contributor in our analysis, operates under national AI development priorities in line with government policy, which may partially explain its concentration of craniofacial and cleft-related AI research linked to large national clinical databases. European contributors, including Italy and Germany, operate within the EU Artificial Intelligence Act framework, which imposes stricter requirements for high-risk AI applications in healthcare, potentially shaping both the types of studies conducted and their governance focus. Whether these regulatory structures directly influence citation impact or research design warrants further investigation.

Beyond geographic trends, our analysis also highlighted a small number of highly productive authors and research groups that have played a central role in advancing AI scholarship within plastic surgery. Investigators affiliated with Peninsula Health, including Rozen and Seth, accounted for a substantial proportion of the most-cited publications in our dataset. Their high publication output is likely influenced by the early adoption of AI-focused research within journals such as the Aesthetic Surgery Journal and Aesthetic Plastic Surgery, which have published a large proportion of recent LLM-related studies and have been receptive to emerging digital health topics. Journals have also played a notable role in shaping the field's development. Several plastic surgery journals have recently created dedicated sections or editorial leadership focused on AI and digital surgery, including initiatives within journals such as the Aesthetic Surgery Journal, Aesthetic Surgery Journal Open Forum, Aesthetic Plastic Surgery, and, more recently, Plastic and Reconstructive Surgery—Global Open. These developments suggest that the growing visibility of AI research within the specialty is driven not only by individual investigators but also by editorial initiatives that encourage the evaluation of emerging technologies. Together, these developments highlight how concentrated authorship networks and supportive editorial platforms have accelerated the growth of AI-related research in plastic surgery.

### Limitations and Future Directions

This study carries several inherent limitations that should be considered. First, the analysis was restricted to the Scopus database, which, while the most comprehensive, necessarily excludes scholarship indexed exclusively in Web of Science, PubMed, or specialty databases. Notably, excluding other language databases, such as the China National Knowledge Infrastructure (CNKI), may lead to a systematic underrepresentation of scholarship from other countries. However, given the nature of previous bibliometric analyses (which limited the analysis to a single database), we identified the most comprehensive database and used it for this analysis. Second, citation counts are subject to temporal bias, as more recently published articles have had less time to accumulate citations, potentially underrepresenting the true scholarly attention directed at high-quality work published in 2024-2025. Third, the analysis was limited to the top 100 most-cited articles, which, although a well-established bibliometric convention, excludes a substantial body of emerging literature that may better reflect current research priorities. Additionally, the ASPS evidence hierarchy should be interpreted with caution, because it was primarily designed for clinical comparative studies and may not cleanly fit AI model development papers, ethics papers, or technical reports. Finally, bibliometric analyses inherently measure scholarly influence rather than clinical validity or methodological rigor; therefore, citation frequency does not necessarily reflect the quality or direct applicability of the reported findings.

Future analyses should consider multi-database search strategies that encompass Web of Science, PubMed, and regional repositories such as the CNKI, thereby yielding a more comprehensive and geographically representative mapping of the global research landscape. Second, removing the restriction of English-language publications would capture contributions from non-English-speaking regions that are currently underrepresented, thereby providing a more accurate reflection of worldwide scholarly activity. Finally, incorporating alternative impact measures, such as Altmetric Attention Scores, social media engagement metrics, and citations to policy documentation, alongside traditional citation counts, would offer a more holistic assessment of both scholarly and public engagement with AI research.

## CONCLUSIONS

This bibliometric analysis provides a structured mapping of the 100 most-cited English-language publications on AI in plastic and reconstructive surgery, revealing a field that has undergone rapid, concentrated growth, particularly following the widespread adoption of LLMs after 2022. Aesthetic surgery, craniofacial surgery, and breast reconstruction emerged as the leading subspecialty domains, potentially driven by the availability of structured imaging datasets and the high clinical demand for precision and reproducibility, whereas patient education and ethical governance emerged as the central thematic priorities. Because the field continues to evolve, future work appears to shift further toward LLM-based applications, suggesting a trajectory in which the academic interest observed here may increasingly translate into clinically integrated, patient-facing tools within plastic surgery practice.

## Supplementary Material

ojag148_Supplementary_Data
