# Supplementary material for: Artificial Intelligence in Plastic Surgery: A Bibliometric and Visual Analysis of the 100 Most-Cited English-Language Publications
Source: Aesthet Surg J Open Forum. 2026 Jul 11;8:ojag148. doi: 10.1093/asjof/ojag148 (PMC13426317; doi:10.1093/asjof/ojag148)
Supplement: ojag148_Supplementary_Data [file ojag148_supplementary_data.zip › Appendix 1.docx]

TITLE-ABS-KEY (“Artificial intelligence” OR “Machine learning” OR “Deep learning” OR “Natural Networks” OR “Neural networks” OR “Natural Language Processing” OR “Generative AI” OR “Chat-based generative pre-trained transformer models” OR “Large language models” OR “Intelligence augmentation” OR “Adaptive learning” OR “Cognitive computing” OR “Reinforcement learning” OR “Reinforcement learning from human feedback” OR “Algorithm” OR “Application programming interface” OR “Chatbot” OR “Machine intelligence” OR “Retrieval Augmented Generation” OR “Explainable machine learning” OR “Explainable AI”)

AND

(“Plastic and reconstructive surgery” OR “Aesthetic surgery” OR “Breast surgery” OR “Craniofacial surgery” OR “Breast augmentation” OR “Augmentation mammaplasty” OR “Abdominoplasty” OR “Cleft lip” OR "Cleft palate" OR “DIEP flap” OR “Latissimus dorsi” OR “Liposuction” OR “Microsurgery” OR “Polydactylyl” OR “Syndactylyl” OR “Rhytidectomy” OR “Face lift” OR “Rhinoplasty” OR “Breast Implants” OR "Tissue expander" OR “Blepharoplasty” OR “Brow lift” OR “Supermicrosurgery”)
