# Supplementary material for: Artificial Intelligence in Plastic Surgery: A Bibliometric and Visual Analysis of the 100 Most-Cited English-Language Publications
Source: Aesthet Surg J Open Forum. 2026 Jul 11;8:ojag148. doi: 10.1093/asjof/ojag148 (PMC13426317; doi:10.1093/asjof/ojag148)
Supplement: ojag148_Supplementary_Data [file ojag148_supplementary_data.zip › Appendix 2.docx]

| Rank | Title | Year | Journal | Citation count (n) |
| --- | --- | --- | --- | --- |
| 1 | Aesthetic Surgery Advice and Counseling from Artificial Intelligence: A Rhinoplasty Consultation with ChatGPT^1^ | 2023 | Aesthetic Plastic Surgery | 139 |
| 2 | Big Data and Machine Learning in Plastic Surgery: A New Frontier in Surgical Innovation^2^ | 2016 | Plastic and Reconstructive Surgery | 115 |
| 3 | Evaluating Chatbot Efficacy for Answering Frequently Asked Questions in Plastic Surgery: A ChatGPT Case Study Focused on Breast Augmentation^3^ | 2023 | Aesthetic Surgery Journal | 97 |
| 4 | Personalized Assessment of Craniosynostosis via Statistical Shape Modeling^4^ | 2014 | Medical Image Analysis | 95 |
| 5 | Expanding Cosmetic Plastic Surgery Research With ChatGPT^5^ | 2023 | Aesthetic Surgery Journal | 86 |
| 6 | Diagnose Parkinson’s Disease and Cleft Lip and Palate using Deep Conventional Neural Networks Evolved by IP-Based Chimp Optimization Algorithm^6^ | 2022 | Biomedical Signal Processing and Control | 65 |
| 7 | Evaluation of Online Artificial Intelligence-Generated Information on Common Hand Procedures^7^ | 2023 | Journal of Hand Surgery | 64 |
| 8 | Machine Learning for Predicting Complications in Head and Neck Microvascular Free Tissue Transfer^8^ | 2020 | Laryngoscope | 50 |
| 9 | Applied Deep Learning in Plastic Surgery: Classifying Rhinoplasty with a Mobile App^9^ | 2020 | Journal of Craniofacial Surgery | 47 |
| 10 | Exploring the Role of a Large Language Model on Carpal Tunnel Syndrome Management: An Observation Study of ChatGPT^10^ | 2023 | Journal of Hand Surgery | 46 |
| 11 | Artificial Intelligence Applications and Ethical Challenges in Oral and Maxillo-facial Cosmetic Surgery: A Narrative Review^11^ | 2023 | Maxillofacial Plastic and Reconstructive Surgery | 44 |
| 12 | Optimizing Ophthalmology Patient Education via ChatBot-Generated Materials: Readability Analysis of AI-Generated Patient Education Materials and The American Society of Ophthalmic Plastic and Reconstructive Surgery Patient Brochures^12^ | 2024 | Ophthalmic Plastic and Reconstructive Surgery | 43 |
| 13 | Clinical Applications of Artificial Intelligence and Machine Learning in Children with Cleft Lip and Palate – A Systematic Review^13^ | 2022 | International Journal of Environmental Research and Public Health | 41 |
| 14 | Automatic Detection of Perforators for Microsurgical Reconstruction^14^ | 2020 | Breast | 41 |
| 15 | Making the Subjective Objective: Machine Learning and Rhinoplasty^15^ | 2020 | Aesthetic Surgery Journal | 41 |
| 16 | Machine Learning Applied to Registry Data: Development of a Patient-Specific Prediction Model for Blood Transfusion Requirements During Craniofacial Surgery Using the Pediatric Craniofacial Perioperative Registry Dataset^16^ | 2021 | Anesthesia and Analgesia | 39 |
| 17 | Using Generative Artificial Intelligence Tools in Cosmetic Surgery: A Study on Rhinoplasty, Facelifts, and Blepharoplasty Procedures^17^ | 2023 | Journal of Clinical Medicine | 37 |
| 18 | Facelift Surgery Turns Back the Clock: Artificial Intelligence and Patient Satisfaction Quantitate Value of Procedure Type and Specific Techniques^18^ | 2021 | Aesthetic Surgery Journal | 36 |
| 19 | Use of Simulation in Plastic Surgery Training^19^ | 2020 | Plastic and Reconstructive Surgery – Global Open | 36 |
| 20 | A Systematic Review of Artificial Intelligence Applications in Plastic Surgery: Looking to the Future^20^ | 2022 | Plastic and Reconstructive Surgery – Global Open | 35 |
| 21 | ChatGPT in Plastic and Reconstructive Surgery^21^ | 2023 | Indian Journal of Plastic Surgery | 35 |
| 22 | HypernasalityNet: Deep Recurrent Neural Network for Automatic Hypernasality Detection^22^ | 2019 | International Journal of Medical Informatics | 34 |
| 23 | Machine Learning to Predict Individual Patient-Reported Outcomes at 2-year Follow-up for Women Undergoing Cancer-Related Mastectomy and Breast Reconstruction (INSPiRED-001)^23^ | 2021 | Breast | 33 |
| 24 | Early Prediction of the Need for Orthognathic Surgery in Patients with Repaired Unilateral Cleft Lip and Palate Using Machine Learning and Longitudinal Lateral Cephalometric Analysis Data^24^ | 2021 | Journal of Craniofacial Surgery | 32 |
| 25 | 3D Morphometric Quantification of Maxillae and Defects for Patients with Unilateral Cleft Palate via Deep Learning-Based CBCT Image Auto-Segmentation^25^ | 2021 | Orthodontics and Craniofacial Research | 32 |
| 26 | Artificial Intelligence Versus Expert Plastic Surgeon: Comparative Study Shows ChatGPT “Wins” Rhinoplasty Consultations: Should We Be Worried?^26^ | 2024 | Facial Plastic Surgery and Aesthetic Medicine | 32 |
| 27 | Large Language Models for Intraoperative Decision Support in Plastic Surgery: A Comparison Between ChatGPT-4 and Gemini^27^ | 2024 | Medicine (Lithuania) | 32 |
| 28 | Reliability of Postoperative Free Flap Monitoring with a Novel Prediction Model Based on Supervised Machine Learning^28^ | 2023 | Plastic and Reconstructive Surgery | 32 |
| 29 | Comparison of Patient Education Materials Generated by Chat Generative Pre-Trained Transformer Versus Experts: An Innovative Way to Increase Readability of Patient Education Materials^29^ | 2023 | Annals of Plastic Surgery | 31 |
| 30 | Artificial Intelligence Language Model Performance for Rapid Intraoperative Queries in Plastic Surgery: ChatGPT and the Deep Inferior Epigastric Perforator Flap^30^ | 2024 | Journal of Clinical Medicine | 31 |
| 31 | The Promise and Pitfalls of AI-Generated Anatomical Images: Evaluating Midjourney for Aesthetic Surgery Applications^31^ | 2024 | Aesthetic Plastic Surgery | 30 |
| 32 | Fully Automated Robot-Assisted Surgery for Mandibular Angle Split Osteotomy^32^ | 2020 | Journal of Craniofacial Surgery | 30 |
| 33 | Artificial Intelligence in Plastic Surgery: Insights from Plastic Surgeons, Education Integration, ChatGPT’s Survey Predictions, and the Path Forward^33^ | 2024 | Plastic and Reconstructive Surgery – Global Open | 28 |
| 34 | Can ChatGPT be the Plastic Surgeon’s New Digital Assistant? A Bibliometric Analysis and Scoping Review of ChatGPT in Plastic Surgery Literature^34^ | 2024 | Aesthetic Plastic Surgery | 27 |
| 35 | An Artificial Intelligence Tool for Image Simulation in Rhinoplasty^35^ | 2022 | Facial Plastic Surgery | 27 |
| 36 | Simulation and Artificial Intelligence in Rhinoplasty: A Systematic Review^36^ | 2022 | Aesthetic Plastic Surgery | 27 |
| 37 | Cleft Prediction Before Birth Using Deep Neural Network^37^ | 2020 | Health Informatics Journal | 27 |
| 38 | Use of Artificial Intelligence in the Advancement of Breast Surgery and Implications for Breast Reconstruction: A Narrative Review^38^ | 2023 | Journal of Clinical Medicine | 26 |
| 39 | Turning Back the Clock: Artificial Intelligence Recognition of Age Reduction after Face-Lift Surgery Correlates with Patient Satisfaction^39^ | 2021 | Plastic and Reconstructive Surgery | 25 |
| 40 | Detection of Baseline Emotion in Brow Lift Patients Using Artificial Intelligence^40^ | 2021 | Aesthetic Plastic Surgery | 24 |
| 41 | Easing the Burden on Caregivers – Applications of Artificial Intelligence for Physicians and Caregivers of Children with Cleft Lip and Palate^41^ | 2025 | Cleft Palate Craniofacial Journal | 24 |
| 42 | Dr. ChatGPT: Utilizing Artificial Intelligence in Surgical Education^42^ | 2024 | Cleft Palate Craniofacial Journal | 24 |
| 43 | Robotics in Plastic Surgery: It’s Here^43^ | 2023 | Plastic and Reconstructive Surgery | 24 |
| 44 | A Comprehensive Evaluation of ChatGPT Consultation Quality for Augmentation Mammoplasty: A Comparative Analysis Between Plastic Surgeons and Laypersons^44^ | 2023 | International Journal of Medical Informatics | 24 |
| 45 | Exploring the Potential of ChatGPT-4 in Responding to Common Questions About Abdominoplasty: An AI-Based Case Study of a Plastic Surgery Consultation^45^ | 2024 | Aesthetic Plastic Surgery | 23 |
| 46 | A Narrative Review of Artificial Intelligence (AI) for Objective Assessment of Aesthetic Endpoints in Plastic Surgery^46^ | 2023 | Aesthetic Plastic Surgery | 22 |
| 47 | Can AI Answer My Questions? Utilizing Artificial Intelligence in the Perioperative Assessment for Abdominoplasty Patients^47^ | 2024 | Aesthetic Plastic Surgery | 20 |
| 48 | Evaluation of Rhinoplasty Information from ChatGPT, Gemini, and Claude for Readability and Accuracy^48^ | 2025 | Aesthetic Plastic Surgery | 20 |
| 49 | Google Search Analysis: What Do People Want to Know About Rhinoplasty and Where Do They Find the Answers?^49^ | 2022 | Facial Plastic Surgery and Aesthetic Medicine | 20 |
| 50 | Utilization of ChatGPT-4 in Plastic and Reconstructive Surgery: A Narrative Review^50^ | 2023 | Plastic and Reconstructive Surgery – Global Open | 20 |
| 51 | Evaluating Artificial Intelligence’s Role in Teaching the Reporting and Interpretation of Computed Tomographic Angiography for Preoperative Planning of the Deep Inferior Epigastric Artery Perforator Flap^51^ | 2024 | Journal of Plastic, Reconstructive & Aesthetic Surgery Open | 19 |
| 52 | Nanotechnology and Artificial Intelligence: An Emerging Paradigm for Postoperative Patient Care^52^ | 2023 | Aesthetic Surgery Journal | 19 |
| 53 | Both Patients and Plastic Surgeons Prefer Artificial Intelligence-Generated Microsurgical Information^53^ | 2024 | Journal of Reconstructive Microsurgery | 19 |
| 54 | Artificial Intelligence Modeling to Predict Periprosthetic Infection and Explantation following Implant-Based Reconstruction^54^ | 2023 | Plastic and Reconstructive Surgery | 18 |
| 55 | Three-Dimensional Facial Soft Tissue Changes After Orthognathic Surgery in Cleft Patients Using Artificial Intelligence-Assisted Landmark Autodigitization^55^ | 2021 | Journal of Craniofacial Surgery | 18 |
| 56 | Unleashing the Power of ChatGPT: Revolutionizing Plastic Surgery and Beyond^56^ | 2023 | Aesthetic Surgery Journal | 18 |
| 57 | ChatGPT and Rhinoplasty Recovery: An Exploration of AI’s Role in Postoperative Guidance^57^ | 2024 | Facial Plastic Surgery | 17 |
| 58 | Harnessing the Power of Artificial Intelligence to Teach Cleft Lip Surgery^58^ | 2022 | Plastic and Reconstructive Surgery – Global Open | 17 |
| 59 | Utility and Comparative Performance of Current Artificial Intelligence Large Language Models as Postoperative Medical Support Chatbots in Aesthetic Surgery^59^ | 2024 | Aesthetic Surgery Journal | 17 |
| 60 | Generating Informed Consent Documents Related to Blepharoplasty Using ChatGPT^60^ | 2024 | Ophthalmic Plastic and Reconstructive Surgery | 16 |
| 61 | Transforming Breast Reconstruction: The Pioneering Role of Artificial Intelligence in Preoperative Planning^61^ | 2023 | Gland Surgery | 16 |
| 62 | Using a New Deep Learning Method for 3D Cephalometry in Patients with Cleft Lip and Palate^62^ | 2023 | Journal of Craniofacial Surgery | 16 |
| 63 | Evaluation of Artificial Intelligence-Generated Responses to Common Plastic Surgery Questions^63^ | 2023 | Plastic and Reconstructive Surgery – Global Open | 16 |
| 64 | Artificial Intelligence for Objectively Measuring Years Regained After Facial Rejuvenation Surgery^64^ | 2023 | American Journal of Otolaryngology – Head and Neck Medicine and Surgery | 16 |
| 65 | The Quality of CLP-Related Information for Patients Provided by ChatGPT^65^ | 2025 | Cleft Palate Craniofacial Journal | 15 |
| 66 | Characterizing Patient Questions Before and After Rhinoplasty on Social Media: A Big Data Approach^66^ | 2021 | Aesthetic Plastic Surgery | 15 |
| 67 | Artificial Intelligence for Rhinoplasty Design in Asian Patients^67^ | 2024 | Aesthetic Plastic Surgery | 15 |
| 68 | Quality of the Information Provided by ChatGPT for Patients in Breast Plastic Surgery: Are We Already In The Future?^68^ | 2024 | Journal of Plastic, Reconstructive & Aesthetic Surgery Open | 15 |
| 69 | Potential Role of Artificial Intelligence in Craniofacial Surgery^69^ | 2021 | Archives of Craniofacial Surgery | 15 |
| 70 | Artificial Intelligence in Plastic Surgery, Where Do We Stand?^70^ | 2024 | Journal of Plastic, Reconstructive & Aesthetic Surgery Open | 14 |
| 71 | ChatGPT for Improving Postoperative Instructions in Multiple Fields of Plastic Surgery^71^ | 2024 | Journal of Plastic, Reconstructive & Aesthetic Surgery | 14 |
| 72 | Integrating AI Into Breast Reconstruction Surgery: Exploring Opportunities, Applications, and Challenges^72^ | 2024 | Plastic Surgery | 14 |
| 73 | A Machine Learning Approach to Predicting Donor Site Complications Following DIEP Flap Harvest^73^ | 2022 | Journal of Reconstructive Microsurgery | 14 |
| 74 | Deep-Learning Systems for Diagnosing Cleft Palate on Panoramic Radiographs in Patients with Cleft Alveolus^74^ | 2023 | Oral Radiology | 14 |
| 75 | AI Assistance in Aesthetic Medicine – A Consensus on Objective Medical Standards^75^ | 2024 | Journal of Cosmetic Dermatology | 14 |
| 76 | Harnessing the Power of Artificial Intelligence in Cleft Lip and Palate: An In-Depth Analysis from Diagnosis to Treatment, A Comprehensive Review^76^ | 2024 | Children | 14 |
| 77 | Management of Dupuytren’s Disease: A Multi-Centric Comparative Analysis Between Experienced Hand Surgeons Versus Artificial Intelligence^77^ | 2025 | Diagnostics | 14 |
| 78 | Perceived Age and Attractiveness Using Facial Recognition Software in Rhinoplasty Patients: A Proof-of-Concept Study^78^ | 2022 | Journal of Craniofacial Surgery | 13 |
| 79 | Modern Machiavelli? The Illusion of ChatGPT-Generated Patient Reviews in Plastic and Aesthetic Surgery Based on 9000 Review Classifications^79^ | 2024 | Journal of Plastic, Reconstructive & Aesthetic Surgery | 13 |
| 80 | Estimating Apparent Age Using Artificial Intelligence: Quantifying the Effect of Blepharoplasty^80^ | 2023 | Journal of Plastic, Reconstructive & Aesthetic Surgery | 13 |
| 81 | Performance of ChatGPT in Answering Clinical Questions on the Practical Guideline of Blepharoptosis^81^ | 2024 | Aesthetic Plastic Surgery | 13 |
| 82 | Empowering Surgeons: Will Artificial Intelligence Change Oral and Maxillofacial Surgery?^82^ | 2025 | International Journal of Oral and Maxillofacial Surgery | 12 |
| 83 | Artificial Intelligence for Patient Support: Assessing Retrieval Augmented Generation for Answering Postoperative Rhinoplasty Questions^83^ | 2025 | Aesthetic Surgery Journal | 12 |
| 84 | Improving Readability and Automating Content Analysis of Plastic Surgery Webpages With ChatGPT^84^ | 2024 | Journal of Surgical Research | 12 |
| 85 | Feasibility of Anomaly Score Detected With Deep Learning in Irradiated Breast Cancer Patients With Reconstruction^85^ | 2022 | npj Digital Medicine | 12 |
| 86 | Artificial Intelligence in Facial Plastic and Reconstructive Surgery: A Systematic Review^86^ | 2024 | Facial Plastic Surgery | 12 |
| 87 | Machine Learning, Deep Learning, Artificial Intelligence and Aesthetic Plastic Surgery: A Qualitative Systematic Review^87^ | 2024 | Aesthetic Plastic Surgery | 12 |
| 88 | The Transformative Role of Artificial Intelligence in Plastic and Reconstructive Surgery: Challenges and Opportunities^88^ | 2025 | Journal of Clinical Medicine | 11 |
| 89 | Turn Your Vision Into Reality – AI-Powered Pre-Operative Outcome Simulation in Rhinoplasty Surgery^89^ | 2024 | Aesthetic Plastic Surgery | 11 |
| 90 | Artificial Intelligence, Genuine Outcome: Analysis of 72 Consecutive Cases of Subfascial Augmented Mastopexy with Smooth Round Implants Supported by P4HB Scaffold^90^ | 2024 | Aesthetic Surgery Journal | 11 |
| 91 | Blepharoptosis Consultation with Artificial Intelligence: Aesthetic Surgery Advice and Counseling from Chat Generative Pre-Trained Transformer (ChatGPT)^91^ | 2024 | Aesthetic Plastic Surgery | 11 |
| 92 | Defining Standard Values for FaceReader Facial Expression Software Output^92^ | 2024 | Aesthetic Plastic Surgery | 11 |
| 93 | Deep Learning-Based Diagnostic System for Velopharyngeal Insufficiency Based on Videofluoroscopy in Patients with Repaired Cleft Palates^93^ | 2023 | Journal of Craniofacial Surgery | 11 |
| 94 | Gene-Gene Interaction Among Cell Adhesion Genes and Risk of Non-Syndromic Cleft Lip With or Without Cleft Palate in Chinese Case-Parent Trios^94^ | 2019 | Molecular Genetics and Genomics Medicine | 11 |
| 95 | Application of Augmented Reality Using Automatic Markerless Registration for Facial Plastic and Reconstructive Surgery^95^ | 2024 | Journal of Cranio-Maxillofacial Surgery | 11 |
| 96 | Automated and Data-Driven Plate Computation for Presurgical Cleft Lip and Palate Treatment^96^ | 2023 | International Journal of Computer Assisted Radiology and Surgery | 10 |
| 97 | Effectiveness of ChatGPT in Identifying and Accurately Guiding Patients in Rhinoplasty Complications^97^ | 2023 | Facial Plastic Surgery | 10 |
| 98 | Future Perspective of Risk Prediction in Aesthetic Surgery: Is Artificial Intelligence Reliable?^98^ | 2024 | Aesthetic Surgery Journal | 9 |
| 99 | Evaluating the Efficacy of Large Language Models in CPT Coding for Craniofacial Surgery: A Comparative Analysis^99^ | 2025 | Journal of Craniofacial Surgery | 9 |
| 100 | Predicting Persistent Opioid Use After Hand Surgery: A Machine Learning Approach^100^ | 2024 | Plastic and Reconstructive Surgery | 9 |

**References:**

1. Xie Y, Seth I, Hunter-Smith DJ, Rozen WM, Ross R, Lee M. Aesthetic Surgery Advice and Counseling from Artificial Intelligence: A Rhinoplasty Consultation with ChatGPT. *Aesth Plast Surg*. 2023;47(5):1985-1993. doi:10.1007/s00266-023-03338-7

2. Kanevsky J, Corban J, Gaster R, Kanevsky A, Lin S, Gilardino M. Big Data and Machine Learning in Plastic Surgery: A New Frontier in Surgical Innovation. *Plastic & Reconstructive Surgery*. 2016;137(5):890e-897e. doi:10.1097/PRS.0000000000002088

3. Seth I, Cox A, Xie Y, et al. Evaluating Chatbot Efficacy for Answering Frequently Asked Questions in Plastic Surgery: A ChatGPT Case Study Focused on Breast Augmentation. *Aesthetic Surgery Journal*. 2023;43(10):1126-1135. doi:10.1093/asj/sjad140

4. Mendoza CS, Safdar N, Okada K, Myers E, Rogers GF, Linguraru MG. Personalized assessment of craniosynostosis via statistical shape modeling. *Medical Image Analysis*. 2014;18(4):635-646. doi:10.1016/j.media.2014.02.008

5. Gupta R, Park JB, Bisht C, et al. Expanding Cosmetic Plastic Surgery Research With ChatGPT. *Aesthetic Surgery Journal*. 2023;43(8):930-937. doi:10.1093/asj/sjad069

6. Chen F, Yang C, Khishe M. Diagnose Parkinson’s disease and cleft lip and palate using deep convolutional neural networks evolved by IP-based chimp optimization algorithm. *Biomedical Signal Processing and Control*. 2022;77:103688. doi:10.1016/j.bspc.2022.103688

7. Crook BS, Park CN, Hurley ET, Richard MJ, Pidgeon TS. Evaluation of Online Artificial Intelligence-Generated Information on Common Hand Procedures. *The Journal of Hand Surgery*. 2023;48(11):1122-1127. doi:10.1016/j.jhsa.2023.08.003

8. Formeister EJ, Baum R, Knott PD, et al. Machine Learning for Predicting Complications in Head and Neck Microvascular Free Tissue Transfer. *The Laryngoscope*. 2020;130(12). doi:10.1002/lary.28508

9. Borsting E, DeSimone R, Ascha M, Ascha M. Applied Deep Learning in Plastic Surgery: Classifying Rhinoplasty With a Mobile App. *Journal of Craniofacial Surgery*. 2020;31(1):102-106. doi:10.1097/SCS.0000000000005905

10. Seth I, Xie Y, Rodwell A, et al. Exploring the Role of a Large Language Model on Carpal Tunnel Syndrome Management: An Observation Study of ChatGPT. *The Journal of Hand Surgery*. 2023;48(10):1025-1033. doi:10.1016/j.jhsa.2023.07.003

11. Rokhshad R, Keyhan SO, Yousefi P. Artificial intelligence applications and ethical challenges in oral and maxillo-facial cosmetic surgery: a narrative review. *Maxillofac Plast Reconstr Surg*. 2023;45(1):14. doi:10.1186/s40902-023-00382-w

12. Eid K, Eid A, Wang D, Raiker RS, Chen S, Nguyen J. Optimizing Ophthalmology Patient Education via ChatBot-Generated Materials: Readability Analysis of AI-Generated Patient Education Materials and The American Society of Ophthalmic Plastic and Reconstructive Surgery Patient Brochures. *Ophthalmic Plastic & Reconstructive Surgery*. 2024;40(2):212-216. doi:10.1097/IOP.0000000000002549

13. Huqh MZU, Abdullah JY, Wong LS, et al. Clinical Applications of Artificial Intelligence and Machine Learning in Children with Cleft Lip and Palate—A Systematic Review. *IJERPH*. 2022;19(17):10860. doi:10.3390/ijerph191710860

14. Mavioso C, Araújo RJ, Oliveira HP, et al. Automatic detection of perforators for microsurgical reconstruction. *The Breast*. 2020;50:19-24. doi:10.1016/j.breast.2020.01.001

15. Dorfman R, Chang I, Saadat S, Roostaeian J. Making the Subjective Objective: Machine Learning and Rhinoplasty. *Aesthetic Surgery Journal*. 2020;40(5):493-498. doi:10.1093/asj/sjz259

16. Jalali A, Lonsdale H, Zamora LV, et al. Machine Learning Applied to Registry Data: Development of a Patient-Specific Prediction Model for Blood Transfusion Requirements During Craniofacial Surgery Using the Pediatric Craniofacial Perioperative Registry Dataset. *Anesthesia & Analgesia*. 2021;132(1):160-171. doi:10.1213/ANE.0000000000004988

17. Lim B, Seth I, Kah S, et al. Using Generative Artificial Intelligence Tools in Cosmetic Surgery: A Study on Rhinoplasty, Facelifts, and Blepharoplasty Procedures. *JCM*. 2023;12(20):6524. doi:10.3390/jcm12206524

18. Gibstein AR, Chen K, Nakfoor B, et al. Facelift Surgery Turns Back the Clock: Artificial Intelligence and Patient Satisfaction Quantitate Value of Procedure Type and Specific Techniques. *Aesthetic Surgery Journal*. 2021;41(9):987-999. doi:10.1093/asj/sjaa238

19. Agrawal N, Turner A, Grome L, et al. Use of Simulation in Plastic Surgery Training. *Plastic and Reconstructive Surgery - Global Open*. 2020;8(7):e2896. doi:10.1097/GOX.0000000000002896

20. Spoer DL, Kiene JM, Dekker PK, et al. A Systematic Review of Artificial Intelligence Applications in Plastic Surgery: Looking to the Future. *Plastic and Reconstructive Surgery - Global Open*. 2022;10(12):e4608. doi:10.1097/GOX.0000000000004608

21. Sharma SC, Ramchandani JP, Thakker A, Lahiri A. ChatGPT in Plastic and Reconstructive Surgery. *Indian J Plast Surg*. 2023;56(04):320-325. doi:10.1055/s-0043-1771514

22. Wang X, Yang S, Tang M, Yin H, Huang H, He L. HypernasalityNet: Deep recurrent neural network for automatic hypernasality detection. *International Journal of Medical Informatics*. 2019;129:1-12. doi:10.1016/j.ijmedinf.2019.05.023

23. Pfob A, Mehrara BJ, Nelson JA, Wilkins EG, Pusic AL, Sidey-Gibbons C. Machine learning to predict individual patient-reported outcomes at 2-year follow-up for women undergoing cancer-related mastectomy and breast reconstruction (INSPiRED-001). *The Breast*. 2021;60:111-122. doi:10.1016/j.breast.2021.09.009

24. Lin G, Kim PJ, Baek SH, Kim HG, Kim SW, Chung JH. Early Prediction of the Need for Orthognathic Surgery in Patients With Repaired Unilateral Cleft Lip and Palate Using Machine Learning and Longitudinal Lateral Cephalometric Analysis Data. *Journal of Craniofacial Surgery*. 2021;32(2):616-620. doi:10.1097/SCS.0000000000006943

25. Wang X, Pastewait M, Wu T, et al. 3D morphometric quantification of maxillae and defects for patients with unilateral cleft palate via deep learning‐based CBCT image auto‐segmentation. *Orthod Craniofacial Res*. 2021;24(S2):108-116. doi:10.1111/ocr.12482

26. Durairaj KK, Baker O, Bertossi D, et al. Artificial Intelligence Versus Expert Plastic Surgeon: Comparative Study Shows ChatGPT “Wins” Rhinoplasty Consultations: Should We Be Worried? *Facial Plastic Surgery & Aesthetic Medicine*. 2024;26(3):270-275. doi:10.1089/fpsam.2023.0224

27. Gomez-Cabello CA, Borna S, Pressman SM, Haider SA, Forte AJ. Large Language Models for Intraoperative Decision Support in Plastic Surgery: A Comparison between ChatGPT-4 and Gemini. *Medicina*. 2024;60(6):957. doi:10.3390/medicina60060957

28. Huang RW, Tsai TY, Hsieh YH, et al. Reliability of Postoperative Free Flap Monitoring with a Novel Prediction Model Based on Supervised Machine Learning. *Plastic & Reconstructive Surgery*. 2023;152(5):943e-952e. doi:10.1097/PRS.0000000000010307

29. Hung YC, Chaker SC, Sigel M, Saad M, Slater ED. Comparison of Patient Education Materials Generated by Chat Generative Pre-Trained Transformer Versus Experts: An Innovative Way to Increase Readability of Patient Education Materials. *Ann Plast Surg*. 2023;91(4):409-412. doi:10.1097/SAP.0000000000003634

30. Atkinson CJ, Seth I, Xie Y, et al. Artificial Intelligence Language Model Performance for Rapid Intraoperative Queries in Plastic Surgery: ChatGPT and the Deep Inferior Epigastric Perforator Flap. *JCM*. 2024;13(3):900. doi:10.3390/jcm13030900

31. Buzzaccarini G, Degliuomini RS, Borin M, et al. The Promise and Pitfalls of AI-Generated Anatomical Images: Evaluating Midjourney for Aesthetic Surgery Applications. *Aesth Plast Surg*. 2024;48(9):1874-1883. doi:10.1007/s00266-023-03826-w

32. Sun M, Chai Y, Chai G, Zheng X. Fully Automatic Robot-Assisted Surgery for Mandibular Angle Split Osteotomy. *Journal of Craniofacial Surgery*. 2020;31(2):336-339. doi:10.1097/SCS.0000000000005587

33. Farid Y, Fernando Botero Gutierrez L, Ortiz S, et al. Artificial Intelligence in Plastic Surgery: Insights from Plastic Surgeons, Education Integration, ChatGPT’s Survey Predictions, and the Path Forward. *Plastic and Reconstructive Surgery - Global Open*. 2024;12(1):e5515. doi:10.1097/GOX.0000000000005515

34. Liu HY, Alessandri-Bonetti M, Arellano JA, Egro FM. Can ChatGPT be the Plastic Surgeon’s New Digital Assistant? A Bibliometric Analysis and Scoping Review of ChatGPT in Plastic Surgery Literature. *Aesth Plast Surg*. 2024;48(8):1644-1652. doi:10.1007/s00266-023-03709-0

35. Chinski H, Lerch R, Tournour D, Chinski L, Caruso D. An Artificial Intelligence Tool for Image Simulation in Rhinoplasty. *Facial Plast Surg*. 2022;38(02):201-206. doi:10.1055/s-0041-1729911

36. Eldaly AS, Avila FR, Torres-Guzman RA, et al. Simulation and Artificial Intelligence in Rhinoplasty: A Systematic Review. *Aesth Plast Surg*. 2022;46(5):2368-2377. doi:10.1007/s00266-022-02883-x

37. Shafi N, Bukhari F, Iqbal W, Almustafa KM, Asif M, Nawaz Z. Cleft prediction before birth using deep neural network. *Health Informatics J*. 2020;26(4):2568-2585. doi:10.1177/1460458220911789

38. Seth I, Bulloch G, Joseph K, Hunter-Smith DJ, Rozen WM. Use of Artificial Intelligence in the Advancement of Breast Surgery and Implications for Breast Reconstruction: A Narrative Review. *JCM*. 2023;12(15):5143. doi:10.3390/jcm12155143

39. Zhang BH, Chen K, Lu SM, et al. Turning Back the Clock: Artificial Intelligence Recognition of Age Reduction after Face-Lift Surgery Correlates with Patient Satisfaction. *Plastic & Reconstructive Surgery*. 2021;148(1):45-54. doi:10.1097/PRS.0000000000008020

40. Boonipat T, Lin J, Bite U. Detection of Baseline Emotion in Brow Lift Patients Using Artificial Intelligence. *Aesth Plast Surg*. 2021;45(6):2742-2748. doi:10.1007/s00266-021-02430-0

41. Chaker SC, Hung YC, Saad M, Golinko MS, Galdyn IA. Easing the Burden on Caregivers- Applications of Artificial Intelligence for Physicians and Caregivers of Children with Cleft Lip and Palate. *The Cleft Palate Craniofacial Journal*. 2025;62(4):574-587. doi:10.1177/10556656231223596

42. Lebhar MS, Velazquez A, Goza S, Hoppe IC. Dr. ChatGPT: Utilizing Artificial Intelligence in Surgical Education. *The Cleft Palate Craniofacial Journal*. 2024;61(12):2067-2073. doi:10.1177/10556656231193966

43. Henn D, Trotsyuk AA, Barrera JA, et al. Robotics in Plastic Surgery: It’s Here. *Plastic & Reconstructive Surgery*. 2023;152(1):239-249. doi:10.1097/PRS.0000000000010270

44. Yun JY, Kim DJ, Lee N, Kim EK. A comprehensive evaluation of ChatGPT consultation quality for augmentation mammoplasty: A comparative analysis between plastic surgeons and laypersons. *International Journal of Medical Informatics*. 2023;179:105219. doi:10.1016/j.ijmedinf.2023.105219

45. Li W, Chen J, Chen F, Liang J, Yu H. Exploring the Potential of ChatGPT-4 in Responding to Common Questions About Abdominoplasty: An AI-Based Case Study of a Plastic Surgery Consultation. *Aesth Plast Surg*. 2024;48(8):1571-1583. doi:10.1007/s00266-023-03660-0

46. Atiyeh B, Emsieh S, Hakim C, Chalhoub R. A Narrative Review of Artificial Intelligence (AI) for Objective Assessment of Aesthetic Endpoints in Plastic Surgery. *Aesth Plast Surg*. 2023;47(6):2862-2873. doi:10.1007/s00266-023-03328-9

47. Lim B, Seth I, Cuomo R, et al. Can AI Answer My Questions? Utilizing Artificial Intelligence in the Perioperative Assessment for Abdominoplasty Patients. *Aesth Plast Surg*. 2024;48(22):4712-4724. doi:10.1007/s00266-024-04157-0

48. Meyer MKR, Kandathil CK, Davis SJ, et al. Evaluation of Rhinoplasty Information from ChatGPT, Gemini, and Claude for Readability and Accuracy. *Aesth Plast Surg*. 2025;49(7):1868-1873. doi:10.1007/s00266-024-04343-0

49. Fassas SN, Krane NA, Zonner JG, Sykes KJ, Kriet JD, Humphrey CD. Google Search Analysis: What Do People Want to Know About Rhinoplasty and Where Do They Find the Answers? *Facial Plastic Surgery & Aesthetic Medicine*. 2022;24(5):363-368. doi:10.1089/fpsam.2021.0100

50. Aljindan FK, Shawosh MH, Altamimi L, Arif S, Mortada H. Utilization of ChatGPT-4 in Plastic and Reconstructive Surgery: A Narrative Review. *Plastic and Reconstructive Surgery - Global Open*. 2023;11(10):e5305. doi:10.1097/GOX.0000000000005305

51. Lim B, Cevik J, Seth I, et al. Evaluating Artificial Intelligence’s Role in Teaching the Reporting and Interpretation of Computed Tomographic Angiography for Preoperative Planning of the Deep Inferior Epigastric Artery Perforator Flap. *JPRAS Open*. 2024;40:273-285. doi:10.1016/j.jpra.2024.03.010

52. Del Vecchio D, Stein MJ, Dayan E, Marte J, Theodorou S. Nanotechnology and Artificial Intelligence: An Emerging Paradigm for Postoperative Patient Care. *Aesthetic Surgery Journal*. 2023;43(7):748-757. doi:10.1093/asj/sjad071

53. Berry CE, Fazilat AZ, Lavin C, et al. Both Patients and Plastic Surgeons Prefer Artificial Intelligence–Generated Microsurgical Information. *J Reconstr Microsurg*. 2024;40(09):657-664. doi:10.1055/a-2273-4163

54. Hassan AM, Biaggi-Ondina A, Asaad M, et al. Artificial Intelligence Modeling to Predict Periprosthetic Infection and Explantation following Implant-Based Reconstruction. *Plastic & Reconstructive Surgery*. 2023;152(5):929-938. doi:10.1097/PRS.0000000000010345

55. Seo J, Yang IH, Choi JY, Lee JH, Baek SH. Three-Dimensional Facial Soft Tissue Changes After Orthognathic Surgery in Cleft Patients Using Artificial Intelligence-Assisted Landmark Autodigitization. *Journal of Craniofacial Surgery*. 2021;32(8):2695-2700. doi:10.1097/SCS.0000000000007712

56. Bassiri-Tehrani B, Cress PE. Unleashing the Power of ChatGPT: Revolutionizing Plastic Surgery and Beyond. *Aesthetic Surgery Journal*. 2023;43(11):1395-1399. doi:10.1093/asj/sjad135

57. Capelleras M, Soto-Galindo GA, Cruellas M, Apaydin F. ChatGPT and Rhinoplasty Recovery: An Exploration of AI’s Role in Postoperative Guidance. *Facial Plast Surg*. 2024;40(05):628-631. doi:10.1055/a-2219-4901

58. Sayadi LR, Hamdan US, Zhangli Q, Vyas RM. Harnessing the Power of Artificial Intelligence to Teach Cleft Lip Surgery. *Plastic and Reconstructive Surgery - Global Open*. 2022;10(7):e4451. doi:10.1097/GOX.0000000000004451

59. Abi-Rafeh J, Henry N, Xu HH, et al. Utility and Comparative Performance of Current Artificial Intelligence Large Language Models as Postoperative Medical Support Chatbots in Aesthetic Surgery. *Aesthetic Surgery Journal*. 2024;44(8):889-896. doi:10.1093/asj/sjae025

60. Shiraishi M, Tomioka Y, Miyakuni A, et al. Generating Informed Consent Documents Related to Blepharoplasty Using ChatGPT. *Ophthalmic Plastic & Reconstructive Surgery*. 2024;40(3):316-320. doi:10.1097/IOP.0000000000002574

61. Cevik J, Seth I, Rozen WM. Transforming breast reconstruction: the pioneering role of artificial intelligence in preoperative planning. *Gland Surg*. 2023;12(9):1271-1275. doi:10.21037/gs-23-265

62. Xu M, Liu B, Luo Z, et al. Using a New Deep Learning Method for 3D Cephalometry in Patients With Cleft Lip and Palate. *Journal of Craniofacial Surgery*. 2023;34(5):1485-1488. doi:10.1097/SCS.0000000000009299

63. Copeland-Halperin LR, O’Brien L, Copeland M. Evaluation of Artificial Intelligence–generated Responses to Common Plastic Surgery Questions. *Plastic and Reconstructive Surgery - Global Open*. 2023;11(8):e5226. doi:10.1097/GOX.0000000000005226

64. Elliott ZT, Bheemreddy A, Fiorella M, et al. Artificial intelligence for objectively measuring years regained after facial rejuvenation surgery. *American Journal of Otolaryngology*. 2023;44(2):103775. doi:10.1016/j.amjoto.2022.103775

65. Duran GS, Yurdakurban E, Topsakal KG. The Quality of CLP-Related Information for Patients Provided by ChatGPT. *The Cleft Palate Craniofacial Journal*. 2025;62(4):588-595. doi:10.1177/10556656231222387

66. Tseng CC, Gao J, Talmor G, Paskhover B. Characterizing Patient Questions Before and After Rhinoplasty on Social Media: A Big Data Approach. *Aesth Plast Surg*. 2021;45(4):1685-1692. doi:10.1007/s00266-021-02203-9

67. Li R, Shu F, Zhen Y, Song Z, An Y, Jiang Y. Artificial Intelligence for Rhinoplasty Design in Asian Patients. *Aesth Plast Surg*. 2024;48(8):1557-1564. doi:10.1007/s00266-023-03534-5

68. Grippaudo FR, Nigrelli S, Patrignani A, Ribuffo D. Quality of the Information provided by ChatGPT for Patients in Breast Plastic Surgery: Are we already in the future? *JPRAS Open*. 2024;40:99-105. doi:10.1016/j.jpra.2024.02.001

69. Ryu JY, Chung HY, Choi KY. Potential role of artificial intelligence in craniofacial surgery. *Arch Craniofac Surg*. 2021;22(5):223-231. doi:10.7181/acfs.2021.00507

70. Kiwan O, Al-Kalbani M, Rafie A, Hijazi Y. Artificial intelligence in plastic surgery, where do we stand? *JPRAS Open*. 2024;42:234-243. doi:10.1016/j.jpra.2024.09.003

71. Zhang A, Li CXR, Piper M, Rose J, Chen K, Lin AY. ChatGPT for improving postoperative instructions in multiple fields of plastic surgery. *Journal of Plastic, Reconstructive & Aesthetic Surgery*. 2024;99:201-208. doi:10.1016/j.bjps.2024.08.065

72. Gorgy A, Xu HH, Hawary HE, Nepon H, Lee J, Vorstenbosch J. Integrating AI into Breast Reconstruction Surgery: Exploring Opportunities, Applications, and Challenges. *Plast Surg (Oakv)*. 2026;34(1):37-46. doi:10.1177/22925503241292349

73. Huang H, Lu Wang M, Chen Y, Chadab TM, Vernice NA, Otterburn DM. A Machine Learning Approach to Predicting Donor Site Complications Following DIEP Flap Harvest. *J Reconstr Microsurg*. 2024;40(01):070-077. doi:10.1055/a-2071-3368

74. Kuwada C, Ariji Y, Kise Y, et al. Deep-learning systems for diagnosing cleft palate on panoramic radiographs in patients with cleft alveolus. *Oral Radiol*. 2023;39(2):349-354. doi:10.1007/s11282-022-00644-9

75. Frank K, Day D, Few J, et al. AI assistance in aesthetic medicine–A consensus on objective medical standards. *J of Cosmetic Dermatology*. 2024;23(12):4110-4115. doi:10.1111/jocd.16481

76. Almoammar KA. Harnessing the Power of Artificial Intelligence in Cleft Lip and Palate: An In-Depth Analysis from Diagnosis to Treatment, a Comprehensive Review. *Children*. 2024;11(2):140. doi:10.3390/children11020140

77. Seth I, Marcaccini G, Lim K, et al. Management of Dupuytren’s Disease: A Multi-Centric Comparative Analysis Between Experienced Hand Surgeons Versus Artificial Intelligence. *Diagnostics*. 2025;15(5):587. doi:10.3390/diagnostics15050587

78. Khetpal S, Peck C, Parsaei Y, et al. Perceived Age and Attractiveness Using Facial Recognition Software in Rhinoplasty Patients: A Proof-of-Concept Study. *Journal of Craniofacial Surgery*. 2022;33(5):1540-1544. doi:10.1097/SCS.0000000000008625

79. Knoedler S, Sofo G, Kern B, et al. Modern Machiavelli? The illusion of ChatGPT-generated patient reviews in plastic and aesthetic surgery based on 9000 review classifications. *Journal of Plastic, Reconstructive & Aesthetic Surgery*. 2024;88:99-108. doi:10.1016/j.bjps.2023.10.119

80. Goodyear K, Saffari PS, Esfandiari M, Baugh S, Rootman DB, Karlin JN. Estimating apparent age using artificial intelligence: Quantifying the effect of blepharoplasty. *Journal of Plastic, Reconstructive & Aesthetic Surgery*. 2023;85:336-343. doi:10.1016/j.bjps.2023.07.017

81. Shiraishi M, Tomioka Y, Miyakuni A, et al. Performance of ChatGPT in Answering Clinical Questions on the Practical Guideline of Blepharoptosis. *Aesth Plast Surg*. 2024;48(13):2389-2398. doi:10.1007/s00266-024-04005-1

82. Sillmann YM, Monteiro JLGC, Eber P, Baggio AMP, Peacock ZS, Guastaldi FPS. Empowering surgeons: will artificial intelligence change oral and maxillofacial surgery? *International Journal of Oral and Maxillofacial Surgery*. 2025;54(2):179-190. doi:10.1016/j.ijom.2024.09.004

83. Genovese A, Prabha S, Borna S, et al. Artificial Intelligence for Patient Support: Assessing Retrieval-Augmented Generation for Answering Postoperative Rhinoplasty Questions. *Aesthetic Surgery Journal*. 2025;45(7):735-744. doi:10.1093/asj/sjaf038

84. Fanning JE, Escobar-Domingo MJ, Foppiani J, et al. Improving Readability and Automating Content Analysis of Plastic Surgery Webpages With ChatGPT. *Journal of Surgical Research*. 2024;299:103-111. doi:10.1016/j.jss.2024.04.006

85. Kim DY, Lee SJ, Kim EK, et al. Feasibility of anomaly score detected with deep learning in irradiated breast cancer patients with reconstruction. *npj Digit Med*. 2022;5(1):125. doi:10.1038/s41746-022-00671-0

86. Espinosa Reyes JA, Puerta Romero M, Cobo R, Heredia N, Solís Ruiz LA, Corredor Zuluaga DA. Artificial Intelligence in Facial Plastic and Reconstructive Surgery: A Systematic Review. *Facial Plast Surg*. 2024;40(05):615-622. doi:10.1055/a-2216-5099

87. Nogueira R, Eguchi M, Kasmirski J, et al. Machine Learning, Deep Learning, Artificial Intelligence and Aesthetic Plastic Surgery: A Qualitative Systematic Review. *Aesth Plast Surg*. 2025;49(1):389-399. doi:10.1007/s00266-024-04421-3

88. Mansoor M, Ibrahim AF. The Transformative Role of Artificial Intelligence in Plastic and Reconstructive Surgery: Challenges and Opportunities. *JCM*. 2025;14(8):2698. doi:10.3390/jcm14082698

89. Knoedler S, Alfertshofer M, Simon S, et al. Turn Your Vision into Reality—AI-Powered Pre-operative Outcome Simulation in Rhinoplasty Surgery. *Aesth Plast Surg*. 2024;48(23):4833-4838. doi:10.1007/s00266-024-04043-9

90. Bistoni G, Sofo F, Cagli B, Buccheri EM, Mallucci P. Artificial Intelligence, Genuine Outcome: Analysis of 72 Consecutive Cases of Subfascial Augmentation Mastopexy With Smooth Round Implants Supported by P4HB Scaffold. *Aesthetic Surgery Journal*. 2024;44(11):1154-1166. doi:10.1093/asj/sjae109

91. Shiraishi M, Tanigawa K, Tomioka Y, et al. Blepharoptosis Consultation with Artificial Intelligence: Aesthetic Surgery Advice and Counseling from Chat Generative Pre-Trained Transformer (ChatGPT). *Aesth Plast Surg*. 2024;48(11):2057-2063. doi:10.1007/s00266-024-04002-4

92. Zhu A, Boonipat T, Cherukuri S, Bite U. Defining Standard Values for FaceReader Facial Expression Software Output. *Aesth Plast Surg*. 2024;48(5):785-792. doi:10.1007/s00266-023-03468-y

93. Ha JH, Lee H, Kwon SM, et al. Deep Learning–Based Diagnostic System for Velopharyngeal Insufficiency Based on Videofluoroscopy in Patients With Repaired Cleft Palates. *Journal of Craniofacial Surgery*. 2023;34(8):2369-2375. doi:10.1097/SCS.0000000000009560

94. Liu D, Wang M, Yuan Y, et al. Gene–gene interaction among cell adhesion genes and risk of nonsyndromic cleft lip with or without cleft palate in Chinese case‐parent trios. *Molec Gen &amp; Gen Med*. 2019;7(10):e00872. doi:10.1002/mgg3.872

95. Kim YC, Park CU, Lee SJ, Jeong WS, Na SW, Choi JW. Application of augmented reality using automatic markerless registration for facial plastic and reconstructive surgery. *Journal of Cranio-Maxillofacial Surgery*. 2024;52(2):246-251. doi:10.1016/j.jcms.2023.12.009

96. Schnabel TN, Gözcü B, Gotardo P, et al. Automated and data-driven plate computation for presurgical cleft lip and palate treatment. *Int J CARS*. 2023;18(6):1119-1125. doi:10.1007/s11548-023-02858-6

97. Soto-Galindo GA, Capelleras M, Cruellas M, Apaydin F. Effectiveness of ChatGPT in Identifying and Accurately Guiding Patients in Rhinoplasty Complications. *Facial Plast Surg*. 2024;40(05):623-627. doi:10.1055/a-2218-6984

98. Duran A, Cortuk O, Ok B. Future Perspective of Risk Prediction in Aesthetic Surgery: Is Artificial Intelligence Reliable? *Aesthetic Surgery Journal*. 2024;44(11):NP839-NP849. doi:10.1093/asj/sjae140

99. Isch EL, Sarikonda A, Sambangi A, et al. Evaluating the Efficacy of Large Language Models in CPT Coding for Craniofacial Surgery: A Comparative Analysis. *Journal of Craniofacial Surgery*. 2025;36(3):831-835. doi:10.1097/SCS.0000000000010575

100. Baxter NB, Ho AZ, Byrd JN, Fernandez AC, Singh K, Chung KC. Predicting Persistent Opioid Use after Hand Surgery: A Machine Learning Approach. *Plastic & Reconstructive Surgery*. 2024;154(3):573-580. doi:10.1097/PRS.0000000000011099
